# Carcass trait, meat yield and quality characteristics of recently-synthesized Woori Heukdon and commercial LYD pigs under identical rearing condition

**DOI:** 10.5713/ab.22.0304

**Published:** 2023-01-10

**Authors:** Van-Ba Hoa, Dong-Heon Song, Ye-Jin Min, Kuk-Hwan Seol, Sun-Moon Kang, Hyun-Wook Kim, Sung-Sil Moon, Soo-Hyun Cho

**Affiliations:** 1 Animal Products Utilization Division, National Institute of Animal Science, RDA, Wanju 55365, Korea; 2 Swine Science Division, National Institute of Animal Science, RDA, Cheonan 31000, Korea; 3 Sunjin Meat Research Center, Ansung 17532, Korea

**Keywords:** Meat Yield, Quality, Sensory Property, Woori Heukdon

## Abstract

**Objective:**

For decades, LYD ([Landrace×Yorkshire] ♀×Duroc ♂) pigs are the most commonly-used commercial breed for meat production in Korea. Recently, due to the increasing demand for premium pork, the National Institute of Animal Science (Korea) has synthesized a novel pig breed named Woori Heukdon (WHD). This study aimed at comparing the carcass traits, meat yield and quality characteristics between the LYD and WHD pigs under identical rearing condition.

**Methods:**

The WHD and LYD pigs (n = 15 each) were reared under identical conditions and fed the same commercial diet until reaching recommended market weight (100 to 120 kg). After slaughter, the carcasses were evaluated for traits and meat yield, and the meat quality was assessed on shoulder butt and belly cuts.

**Results:**

Although no significant differences (p>0.05) occurred in slaughter weight between two pig types, WHD had a lower meat yield (by about 6 kg corresponding to approximately 7%) compared to the LYD pigs (p<0.05). The WHD had a higher fat content (by 4.26% and 13.52% in the shoulder butt and belly, respectively) compared to those of LYD pigs (p<0.05). The WHD meat showed a lower cooking loss and higher a* (redness) value (p<0.05). The WHD belly had a significantly (p<0.05) higher oleic acid content and concentrations of nonanal, octanal and decanal associated with fatty odor while, the LYD meat had a higher number of pyrazines associated with roasty odor. Regarding sensory quality, higher flavor, juiciness, and acceptability higher scores were given for the WHD meat than for the LYD meat (p<0.05).

**Conclusion:**

Under identical rearing conditions the WHD exhibited a better meat quality and sensory properties. However, the use of this diet resulted in a high fat level that may be associated with high trimming loss for the WHD.

## INTRODUCTION

In recent years, there is an increasing demand in Korea for premium pork such as Korean native black and imported Spanish Iberico pork, despite its price being approximately 30% to 40% more expensive than other commercial pork [1,2]. The Korea pig industry, therefore, has been partly shifting from production of greater pork yield by focusing on both quantity and quality, to meet this increasing demand. In this context, the National Institute of Animal Science (Korea) has recently developed a novel pig breed named “Woori-Heukdon” (WHD), which is synthesized by crossbreeding between Duroc sow with Korean native black pig (KNP) sire [2]. In 2015, it has been registered as a novel pig breed in the Food and Agriculture Organization Domestic Animal Diversity-Information System. The WHD is expected to display the genetic merits of both KNP and commercial Duroc pig breeds. The KNP is known as an indigenous porcine breed which has been widely raised in Korea since 1970’s [3]. The KNP is characterized by its distinctive short and black coat color, slower growth rate and lighter carcass but stronger tolerance of disease compared to modern and genetically improved breeds [4]. In term of meat quality, the KNP shows a superior meat quality such as; more reddish in color, whiter fat, higher marbling degree and better eating quality than those from other commercial pig breeds [4–7]. While, the Duroc pig is usually exploited as a sire breed, and for meat production purposes owing to its fast-growing rate and better feed conversation ratio etc. [8]

In the modern intensive systems, feed expense accounts for about 60% to 70% of the total cost of pork production, in which the energy content alone represents 50% of the total cost [9]. After consuming, the energy released from carbohydrates, fat and protein in feed is needed for the biosynthetic processes (e.g., proteins, bones, and lipids), maintenance, active ion transport and mechanical work [10]. However, the amount of dietary nutrients/energy level required for the protein and fat deposition widely varies not only depending on the growing or finishing phase [11,12] but also the breed [13]. Providing an improper diet may result in profit loss, environmental pollution, and poor meat quality [9,13,14].

Fat is well recognized as an important component for the technological (e.g., texture and water holding capacity) and eating qualities (e.g., juiciness and flavor) of pork [15–17]. However, an excessive fat level in pork may be associated with high risk of rejection by consumers [18]. The fat deposition is mainly affected by diet and breed [13,19]. Till now, no specific dietary formulation has been designed for the WHD. Currently, the diet and regimes for the WHD follows the commercial production conditions that are the same as those used for the other commercial pig breeds. However, how this diet affects carcass composition, meat yield and quality properties or whether it is suitable for the WHD pig still remains unknown. More to the point, the scientific information regarding the meat quality properties of WHD is scarce, and no research has compared the meat quality of the WHD with commercial pigs. Thus, the aim of this study was to compare the meat yield, chemical composition, technological and eating qualities of the WHD with commercial (Landrace×Yorkshire) ♀×Duroc ♂ (LYD) pigs under identical production conditions.

## MATERIALS AND METHODS

### Animal care

The animal protocols used in the present study were reviewed and approved by the Institutional Animal Care and Use Committee (IACUC) at National Institute of Animal Science (Approval No. NIAS 20001992).

### Animals

A total of thirty same age piglets including: WHD (n = 15) and commercial LYD (n = 15) were used in the present investigation. The WHD was synthesized by crossbreeding between Duroc sow (62.5%)×KNP sire (37.55%) at the experimental pig farm of National Institute of Animal Science (Korea). All the pigs were housed individually in pens and received the same diet (Table 1). During the experimental period, all the animals had free access to the feed via a steel feeder and water via a nipple drinker. The pigs were harvested when reaching the recommended market weight (100 to 120 kg) [20]. The pigs were transported from the experimental farm to a slaughterhouse (Jeonju, Korea) with the transporting time of about 2 h, and were then laired in pens for 6 to 8 h with full access to water.

### Slaughter, meat yield measurement and sampling

The pigs were slaughtered in different batches due to the growth rate difference. The animals were electrically stunned and slaughtered following the commercial process. The carcasses were split and chilled at 2°C±2°C overnight in a chilling room. The next morning, carcasses were weighed to determine dressing percentage (calculated as percentage of cold carcass weight to slaughter weight), and then fabricated into 7 primal cuts including: shoulder butt, tenderloin, loin, belly, hind and fore legs, and shoulder ribs, according to the Korean Pork Cutting Specification [21]. After removing bone, skin, and visual fats (subcutaneous and intermuscular fat tissues), their weights were recorded to determine the meat yield for the cuts. The total meat yield was calculated by summing up the meat weights of all the cuts.

Following the meat yield measurement, all shoulder butt and belly cuts (n = 15 for each breed and cut type) were collected from the left side of carcasses, and immediately used for meat quality analysis. Each the sample was prepared into sub-samples depending on the analysis. Due to the complicated structure with multiple muscles and intermuscular fat layers, the sampling manner was fixed for all the samples as shown in our previous study [22]. The physicochemical quality (proximate composition, pH, color, and cooking loss) was analyzed immediately after sampling (the day after slaughter), and the subsamples for fatty acid, volatile aroma and sensory evaluation were vacuum-packaged and stored at −20°C until use.

### Physicochemical quality measurement

*pH*: The muscle pH was measured by inserting the solid-state probe of a pH meter (pH*K 21; NWK-Technology GmbH, Kaufering, Germany) into 3 and 9 different points on transverse cut surface of each shoulder butt and belly, respectively. Before using, the device was calibrated using standard buffers at pH 4.0 and 7.0.

*Color and cooking loss*: Both the color and cooking loss were measured on same steaks (1 and 3 steaks per shoulder butt and belly, respectively with 3-cm thickness and weight of 200 g) of each sample. The color values (L*, a*, b*, Chroma and hue angle) were measured on 3 and 9 different points on the transverse cut surface of each shoulder butt and belly, respectively after a 30-min exposure to air (blooming) at 4°C using a Minolta Chroma Meter CR-400 with a D65 illuminant*C and 2° observer (Minolta Camera, Osaka, Japan). Before using, the device was calibrated with a standard white plate. Following the color measurement, the weight of each slice was recorded and then placed into plastic bags, sealed, and immersed in a pre-heated 72°C water bath until a core temperature of 70°C was reached. After cooking, the samples were blotted dry with paper towels and their weights were again recorded to determine the cooking loss that was calculated by pre-cooking weight minus post-cooking weight divided pre-cooking weight and multiplied by 100.

### Proximate composition

The proximate composition was determined using a Food Scan Lab 78810 (Foss Tecator Co., Ltd., Hillerod, Denmark) as described in our previous study [17]. Briefly, after chopping and grinding, aliquots of 200 g meat sample was applied on a round sample dish and loaded on the chamber of device. The samples were then analyzed for moisture, protein, fat, and collagen. Each the sample was determined in duplicate.

### Fatty acids

For the fatty acid analysis, total lipids were extracted using a solvent mixture: chloroform: methanol in a ratio of 2:1 (v/v) as described in our previous study [23]. Briefly, each sample (10 g) was homogenized with 150 mL of the solvent mixture at 300×g for 3 min, and the homogenate was then filtered with a Whatman filter paper. Approximately 20 g of Na_2_SO_4_ was added into the filtrate and thoroughly mixed for 1 min. The upper layer containing lipids was collected and transferred into an Erlenmeyer flask, which was then dried at 55°C using a rotary evaporator. The obtained lipids were reconstituted with 1 mL tricosanoic acid and 1 mL of 0.5 N NaOH. The samples were then analyzed using a gas chromatography/flame ionization detector (GC-FID; Varian Technologies, Palo Alto, CA, USA) equipped with a capillary column (30 m×0.25 mm×0.25 μm film thickness; Supelco, Bellefonte, PA, USA). The GC-FID condition used was same as those described by Hoa et al [23]. Fatty acids were expressed as relative percent (%) of total fatty acids analyzed.

### Aroma volatiles

The solid phase microextraction (SPME) technique was used to extract the volatile profiles in the headspace of cooked meat samples. The sample preparation, volatiles extraction and analysis were performed following the protocol as described in our previous study [17]. Briefly, the samples were ground and cooked at around 180°C on an open tin-coated grill for about 1 min. Next, the cooked samples (2.0 g each) were weighed, placed into 20-mL headspace vial, and capped with PTFE-faced silicone septum. One microliter of 2-methyl-3-heptanone (0.816 mg/mL) was also added as internal standard. The extraction of volatiles was performed at 60°C for 45 min using a 75-μm carboxen-polydimethylsiloxane fiber (Supelco, USA) connected with the SPME auto-sampler (model: PAL RSI 85). The extracted volatiles were analyzed using a gas chromatography (model: 8890 GC system) and mass spectrophotometry (5977B MS; Agilent Technologies, Santa Clara, CA, USA) equipped with capillary column (30 m×0.25 mm×0.25 μm film thickness; Agilent J & W Scientific, Folcom, CA, USA). The GC/MS condition set was same as those described in the above-cited reference [17]. The volatile aromas were identified by comparing their mass spectra with those present in the Wiley registry library (Agilent Technologies, USA) and/or by comparing their retention times with those of external standards which were run under the same GC/MS condition. The quantification (μg/g) of the identified compounds was done by comparison of their peak areas with that of the internal standard.

### Sensory evaluation

The sensory analysis was performed using the protocol as described in our previous study [17]. Six members of a panel, consisting of institutional staff (who often participate in the sensory tests of meat, with at least 1-year experience) was used. Prior to use, the frozen vacuum-packaged samples were defrosted for about 2 h at 4°C. Samples were manually cut into 5-mm thick slices which were then made into 7 pieces (30×30 mm), placed on dishes, and coded with random numbers. Out of them, one piece was used for overall color evaluation and the rests were used for tasting. The samples were roasted on an open tin-coated grill for 3 min and turned at the start of browning. Immediately after cooking, each cooked piece was placed on individual paper dish and served to the panelists. The panelists tasted and evaluated for the tenderness, flavor, juiciness, and overall acceptance using a 7-point hedonic scale (7 = extremely like; 6 = like very much; 5 = like moderately; 4 = neither like nor dislike; 3 = dislike moderately; 2 = dislike very much; and 1 = dislike extremely) as described by Meilgaard et al [24]. The color was analyzed 30 min after cutting; a piece of each meat sample was placed on while paper dish, passed to the panelists who viewed for about 5 s and rated using the 7-point scale as above described. A total of 3 sessions with 10 samples each were performed, and each sample was evaluated by 6 panelists. The panelists were provided with drinking water and unsalted crackers to refresh their palate between samples. All sensory sessions were performed in the sensory panel booth room equipped with white lighting.

### Statistical analysis

Data were analyzed using the SAS Enterprise software (version 7.1; SAS Inst. Inc., Cary, NY, USA). In the statistical model, the general linear model procedure was used in which the pig type was considered as a fixed effect, and the carcass traits, meat quality, fatty acids, volatile aromas and sensory attributes were considered as dependent variables. The mean difference was compared using t-test. A probability value of p<0.05 was considered for statistically significant difference in all the tests. Data was presented as means±standard.

## RESULTS AND DISCUSSION

### Carcass traits and meat yield

The carcass traits and meat yield of LYD and WHD under identical raising conditions are presented in Table 2. To reach the recommend slaughter weight of 100 to 120 kg [20], the raising time for the LYD and WHD pigs was around 180 and 210 days, respectively. Though no significant (p>0.05) differences occurred in slaughter weight and carcass weight between two pig types, the WHD showed a higher dressing percentage compared to the WHD (p<0.05). Regarding the meat yield, the LYD had a higher weight for almost all primal cuts (e.g., tenderloin, loin, high leg, and rib). As a result, the total meat yield was about 6 kg higher in the LYD than in the WHD, which is corresponding to approximately 7%. This may be attributed due to a higher trimming loss by meat by-products (e.g., skin, bone, subcutaneous and intermuscular fat) in the WHD. Similar to the current finding, Cho et al [25] reported that the commercial LYD pigs had a higher meat yield compared to other pig breeds.

### Proximate composition and meat quality

**Figure 1:**
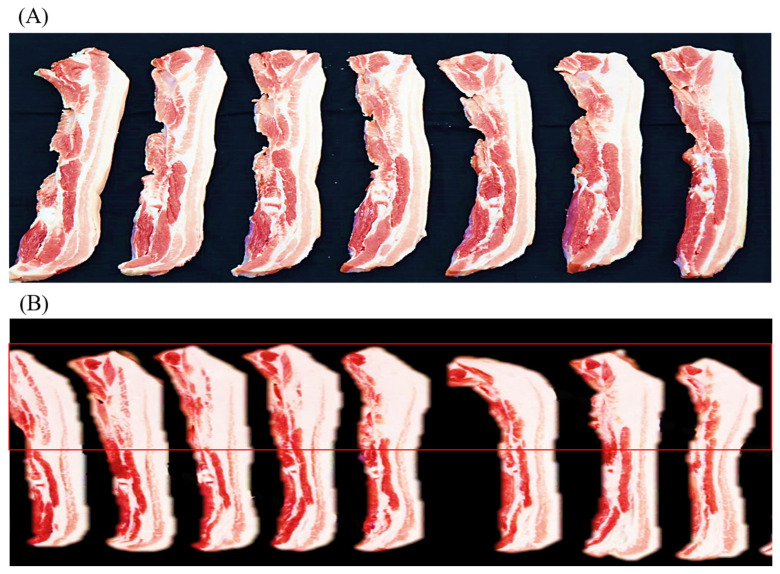
Representative images showing the transverse cut surface of belly slices from 4th –9th rib: Commercial LYD pig (A), and recently synthesized Woori Heukdon (B). LYD, commercial ([Landrace×Yorkshire] ♀×Duroc ♂) pig.

The proximate composition and meat quality traits of the LYD and WHD are presented in Table 3. Regarding the proximate composition, the pig type appeared to affect almost all the chemical composition including moisture, fat, protein, and protein of the meat. The WHD had a higher fat content in the shoulder butt (by 4.26%) and belly (by 13.52%) compared to those of commercial LYD pig (p<0.05). Compared to the commercial LYD, the WHD belly (at the dorsal area from 4th to 9th rib) showed relatively thicker layers of subcutaneous and intermuscular fat as shown in Figure 1. This higher fat content resulted in a lower protein and moisture content in the WHD meat compared to the LYD. Similar to our results, Ali et al [26] and Kim et al [27] found that indigenous pig breeds and their crossbred pigs generally have a higher fat content compared to the other commercial fast-growing pig breeds. Most available research found that commercial pig breeds (e.g., Duroc, Landrace, Yorkshire, and LYD etc.) have a fat range of 20% to 25% and 28% to 33% in shoulder butt and belly cut, respectively [17,28]. Compared to the results reported in these studies, the fat level in the WHD belly was higher (by over 10%). This could be attributed to the genetic background differences that affected the digestibility and absorption of dietary fat and/or De Novo fat synthesis [29]. The adipose tissue or fat tissue is mainly deposited in visceral, subcutaneous, intramuscular, and intermuscular depots [29]. In the present study, both the shoulder butt and belly cuts were fabricated (skinned and trimmed of a relatively thin layer of subcutaneous fat) according to the Korean Pork Cutting Specification [21], their fat content, therefore, is comprised of subcutaneous, intramuscular, and intermuscular fat depots. The adipose tissues may be deposited in pork carcasses from diet or from the De Novo fat synthesis mechanism [14,29]. Researchers have reported [19,30] that genetic background plays an important role in lipogenesis; a higher expression of genes (e.g., acetyl-CoA carboxylase alpha, adiponectin, diacylglycerol acyltransferase 2 and leptin receptor etc.) is involved in the lipogenesis and lipid metabolism in local pig breeds compared to the commercial breeds. Furthermore, the genetic background has also been reported to affect the rate of lipogenesis in the De Novo mechanism involving the converting of glucose into triglycerides [31]. Fat, oil, and carbohydrate are important ingredients in pig’s diet because of their high energy value. In the feeding diet, the carbohydrate intake-derived glucose is taken by adipocytes through insulin-stimulated glucose transporter type-4 [19]. Contrasting to the slow-growing pig breeds, the fast-growing breeds can utilize diets containing high protein and carbohydrates without becoming fat [32]. Based on our results, it may be recommended that an adjustment of dietary energy level (e.g., lowering carbohydrates content) to reduce the excessive glucose is needed to reduce the De Novo fat synthesis and fat deposition in the WHD.

Regarding the meat quality, the pig type appeared to affect almost all traits examined. Particularly, LYD shoulder butt showed a higher pH value compared to the WHD (p<0.05). The rate and extent of postmortem pH decline in pork have been reported to be markedly affected by pre-slaughter (e.g., stress caused by fasting, loading and transport etc., and feeding regime or genetics) and post-slaughter (e.g., chilling rate etc.) factors [33–35]. In the present study we have kept all the aforementioned factors constant for both the pig types. Therefore, the difference in pH result between the pig types might be a genetic effect. In both cuts, the WHD had a lower cooking loss (by 3.31% and 6.04%, in the belly and shoulder butt, respectively) compared to those of LYD (p<0.05), this may be related to their higher fat and lower moisture content. Because fat and moisture are negatively correlated with each other; meat containing higher fat usually has a better water holding capacity [17].

Color is well recognized as the first visual trait reflecting the freshness and wholesomeness of meat [36]. As expected, the WHD meat exhibited a higher a* value (p<0.05), indicating a redder color compared to the commercial LYD meat. This could be attributed to the higher level of muscular protein pigments (e.g., myoglobin) in the WHD meat [36]. Consistent with our results, other studies have also found a redder color in meat from pigs crossbred with indigenous breeds compared to the commercial breeds [8,26,27].

### Fatty acid composition

The fatty acid composition of belly cut from the LYD and WHD are presented in Table 4. With exception of C18:0, C18:1n-7, and C18:2n-6, the pig type affected all the fatty acids identified. Amongst, C18:1-9 (oleic acid) is the most predominant monounsaturated fatty acid (MUFA) whose level was about 4% higher in the WHD compared to the LYD. The C18:1n-9 content has been found to be positively correlated to eating quality, particularly the flavor intensity [37]. As a result, the WHD had higher total MUFA as well as unsaturated fatty acids (UFA) contents compared to the commercial LYD (p<0.05). The mechanism underlining this phenomenon may be related to: i) the better digestion and absorption of fatty acids from the diet at the duodenum [38], and/or ii) a higher degree of fatty acids synthesis in the De Novo pathway with a higher converting rate of glucose into palmitate which is then elongated to produce oleic acid in the WHD [19]. Phospholipids comprising mainly polyunsaturated fatty acids (PUFA), that are membrane components and less affected by breed or feeding diet. In the present study, the total PUFAs were not different between the two pig types (p>0.05). Our results agreed with Wood et al [32] and Kim et al [27], who also reported a significant effect of breed on oleic acid and MUFA content in pork. Furthermore, fatty acid profiles also reflect the nutritional value of meat, which affects the human health. According to the recommendation by Department of Health [39] for a healthy diet, the PUFA/SFA ratio should be >0.4 and the n-6/n-3 PUFA should be <4.0. Both the pork types showed a higher n-6/n-3 and lower PUFA/SFA value than the recommended values. However, the WHD presented a lower n-6/n-3 value compared to the LYD pig (p<0.05).

### Volatile aroma composition

The concentration of volatile aromas in the cooked belly samples from the two pig types are presented in Table 5. Volatile aromas are generated during cooking via the thermal oxidation of fatty acids, Mallard reaction of amino acids and reducing sugar, and their interaction products are the most important compounds contributing to the development of cooked meat flavor [40]. A total of 40 compounds including 21 aldehydes, 6 alcohols, 5 nitrogen-and sulfur-containing compounds and 8 hydrocarbons were identified. The WHD belly showed a higher number of volatile aromas (31 compounds) compared to that of LYD (28 compounds). In general, almost all these compounds have been reported for cooked pork in literatures [17,41]. In both the pork types, aldehydes were the most predominant class of volatile compounds. Aldehydes associated with pleasant odors (e.g., fatty, and fruity) [40,42], are mostly generated from the thermal oxidation of unsaturated fatty acids [40]. Therefore, a small change in fatty acid composition of meat would result in an alteration to the volatile aromas during cooking, which influences the flavor intensity of cooked meat [43]. Out of the aldehydes, heptanal, octanal, nonanal and decanal associated with desirable flavor (e.g., fatty odor) [40,41], were higher in the WHD compared to the LYD (p<0.05). Research conducted to demonstrate the formation pathways of aromatic compounds in cooked meat has shown that these 4 aldehydes are produced from the thermal oxidation of oleic acid [44]. This result may be related to the higher level of oleic acid in the WHD meat (Table 4). Other aldehydes such as E, E,2,4-nonadienal, E, E,2,4-decadienal, E-decanal and 2-undecenal were not found in the LYD meat. These compounds have been reported to be formed from the thermal oxidation of C18:3n-3 [44]. Thus, the absence of these compounds may be related to the significantly lower level of the C18:3n-3 in the LYD meat (Table 4). Due to the low odor detection threshold, alcohols significantly contribute to the cooked meat flavor [40]. We observed that 1-penten-3-ol and 4-amino-1-hexanol were only found in the LYD while, 1-hexanol was only found in the WHD meat. The absences of these alcohols in the meat samples could be related to a low level of their corresponding flavor precursor (e.g., fatty acids and amino acids). Nitrogen- containing compounds such as pyrazines are known to be the important products of Mallard reaction, contributing to the roasty odors of cooked meat [40]. It was noted that all 3 nitrogen- containing compounds (2,5-dimethylpyrazine, 4-methylthiazole and 2-ethyl-3,5-dimethylpyrazine) were not found in the WHD whereas, they were found in the LYD meat, this could be related to its higher protein content (Table 3). Overall, with higher levels of oleic acid-derived compounds the WHD meat may be associated with higher intensity fatty odor while, the LYD meat may be associated with higher intensity roasty odor due the presence of pyrazines.

### Sensory properties

The mean value for sensory attributes of shoulder butt and belly from two pork types are presented in Table 6. It was observed that the pork type appeared to affect all the sensory traits. On a 7-point hedonic scale, the panelists gave significantly (p<0.05) higher color, flavor, juiciness, and tenderness scores for both cuts from WHD than for the LYD. Aligning with the present results, Choi et al [25], and Kim and Kim [7] also reported higher sensory scores for pork from indigenous breeds (e.g., KNP) or their crossbred pigs. Regarding the higher flavor score given for the WHD meat, this may be related to its higher oleic acid level (Table 4) and higher amount of oleic acid-derived volatile aromas associated with fatty odor notes (Table 5) [17,33,36]. The panelists also gave a higher overall acceptance score for both cuts of WHD compared to the LYD (p<0.05), this could be associated with their synergistic effects of the higher color, flavor and juiciness scores.

## CONCLUSION

This study for the first time, compared the meat yield and quality properties between the commercial and recently synthesized WHD pigs under identical rearing condition. Although both the pig types were harvested at the similar body weight, the commercial LYD had a significantly higher meat yield compared to the WHD. Noticeably, the WHD had a higher fat content (by 4.26% and 13.52% in the shoulder butt and belly, respectively) compared to those of commercial LYD pig, which may result in more trimming loss in the WHD. The WHD meat exhibited a better technological quality such as higher water holding capacity and redder color. The WHD meat had a higher concentration of oleic acid-derived volatile aromas associated with fatty odor while, the LYD meat had a higher number of pyrazines associated with roasty odor. The panelists also gave higher scores for all sensory traits in both cuts from WHD. Based on the findings of this study, it is recommended that development of a special feed formation is necessary to reduce the subcutaneous fat and increase the intramuscular fat deposition in the WHD.

## Figures and Tables

**Table 1:** Chemical composition of diets

Chemical composition	Growth stage			

	25 to kg	50 to kg	75 to 100 kg	100 kg to slaughter
Metabolic energy (Kcal/kg)	3,300	3,300	3,300	3,300
Crude protein (%)	18.00	17.00	15.50	14.34
Calcium (%)	0.66	0.59	0.52	0.48
Phosphorus (%)	0.31	0.27	0.24	0.22
Lysine (%)	0.98	0.85	0.73	0.64
Methionine (%)	0.30	0.24	0.21	0.20
Threonine (%)	0.59	0.52	0.46	0.42

**Table 2:** Carcass trait and meat yield of LYD and WHD under identical feeding conditions

Items	LYD	WHD
Carcass traits		
Slaughter weight (kg)	105.37±6.92	103.10±14.45
Cold carcass weight (kg)	78.62±5.89	78.55±11.35
Dressing (%)	74.59±1.71^b^	76.16±1.94^a^
Primal cut	--------- Meat yield in weight (kg) ---------	
Tenderloin	1.17±0.14^a^	0.98±0.16^b^
Loin	7.89±0.73^a^	6.15±1.12^b^
Shoulder butt	4.52±0.38	4.34±0.83
Picnic	9.42±1.06	8.60±1.53
Hind leg	17.42±1.30^a^	14.47±2.47^b^
Belly	12.54±1.36	13.08±1.84
Rib	2.95±0.35^a^	2.01±0.61^b^
Total meat	55.91±4.02^a^	49.63±8.15^b^
Primal cut	------- Meat yield in percentage (%) -------	
Tenderloin	1.23±0.14	1.24±0.12
Loin	8.34±1.05	7.81±0.61
Shoulder butt	4.81±0.77^b^	5.50±0.43^a^
Picnic	9.95±1.31^b^	10.91±0.74^a^
Hind leg	18.48±2.66	18.37±0.92
Belly	13.32±2.31^b^	16.68±0.81^a^
Rib	3.14±0.62^a^	2.51±0.46^b^
Total meat	70.06±2.01^a^	63.02±1.93^b^

WHD, Woori Heukdon; LYD, commercial ([Landrace×Yorkshire] ♀×Duroc ♂) pig.

a,b Means within a row with different superscripts are significantly different (p<0.05).

**Table 3:** Proximate composition and technological quality traits of shoulder butt and belly of WHD and LYD pigs under identical feeding conditions

Items	LYD	WHD	LYD	WHD
	
	Shoulder butt		Belly	
Proximate composition				
Moisture (%)	63.45±6.28	60.81±4.52	53.31±8.12^a^	43.36±7.32^b^
Fat (%)	18.28±8.92^b^	22.51±6.12^a^	30.79±10.89^b^	44.31±8.70^a^
Protein (%)	1 8.57±2.93^a^	16.74±1.37^b^	16.58±2.86^a^	13.12±1.64^b^
Collagen (%)	2.31±1.52	1.81±0.23	2.66±1.84^a^	1.83±0.39^b^
Technological quality				
pH	6.04±0.26^a^	5.83±0.17^b^	5.87±0.15	5.85±0.14
Cooking loss (%)	24.09±1.17^a^	20.78±1.49^b^	16.91±2.03^a^	10.87±1.68^b^
Color traits				
L* (lightness)	48.75±5.15	47.97±2.60	59.00±8.82^a^	47.75±2.61^b^
a* (redness)	12.35±1.93^b^	14.16±1.66^a^	9.89±3.53^b^	13.79±1.92^a^
b* (yellowness)	6.04±1.86	6.52±1.33	6.66±1.54	6.34±1.36
Chroma	13.80±2.38^b^	15.61±1.98^a^	12.07±3.37^b^	15.20±2.24^a^
Hue angle	25.68±5.18	24.58±2.77	35.79±10.10^a^	24.55±2.67^b^

WHD, Woori Heukdon; LYD, commercial ([Landrace×Yorkshire] ♀×Duroc ♂) pig.

a,b Means in the same primal cut within a row with different superscripts are significantly different (p<0.05).

**Table 4:** Relative percentage of fatty acid profiles of WHD and LYD belly cut under identical feeding conditions

Item	LYD	WHD
C14:0	1.84±0.70^a^	1.36±0.15^b^
C16:0	30.27±1.77^a^	27.76±2.13^b^
C16:1n7	2.34±0.40^a^	1.64±0.58^b^
C18:0	13.43±0.86	13.12±1.86
C18:1n9	37.91±2.19^b^	41.56±3.16^a^
C18:1n7	0.05±0.00	0.05±0.01
C18:2n6	12.52±1.94	12.99±1.68
C18:3n6	0.01±0.00^b^	0.02±0.01^a^
C18:3n3	0.56±0.10^b^	0.66±0.12^a^
C20:1n9	0.78±0.11^a^	0.64±0.14^b^
C20:4n6	0.20±0.06^a^	0.15±0.03^b^
SFA	45.54±1.59^a^	42.23±2.56^b^
UFA	54.46±1.59^b^	57.77±2.56^a^
MUFA	41.08±2.26^b^	43.90±2.76^a^
PUFA	13.39±2.07	13.87±1.79
n3	0.56±0.10	0.66±0.12
n6	12.82±1.98	13.21±1.70
n6/n3	23.06±2.37^b^	20.35±2.50^a^
MUFA/SFA	0.90±0.07^b^	1.05±0.12^a^
PUFA/SFA	0.29±0.05	0.33±0.05

WHD, Woori Heukdon; LYD, commercial ([Landrace×Yorkshire] ♀×Duroc ♂) pig; SFA, saturated fatty acids; UFA, unsaturated fatty acids; MUFA, monounsaturated fatty acids; PUFA, poly unsaturated fatty acids.

a,b Means within a row with different superscripts are significantly different (p<0.05).

**Table 5:** Concentration (μg/g) of volatile aromas in WHD and LYD belly cut under identical feeding conditions

Compounds	Retention time (min)	LYD	WHD	Identification method
Aldehydes				
Propanal	1.723	0.05±0.01	ND	MS+STD
2-methyl propanal	1.8778	ND	0.01±0.00	MS
Butanal	2.0634	ND	0.00±0.00	MS+STD
2-ethyl-hexanal	2.167	0.02±0.01	ND	MS
Butanal, 3-methyl-	2.72	0.03±0.02^a^	0.02±0.01^b^	MS+STD
Butanal, 2-methyl-	2.829	0.06±0.03^a^	0.02±0.01^b^	MS+STD
Pentanal	3.154	ND	0.27±0.10	MS+STD
Hexanal	6.121	2.81±0.26^b^	3.87±1.46^a^	MS+STD
Heptanal	9.261	0.15±0.03^b^	0.20±0.05^a^	MS+STD
E,2-Heptenal	10.755	0.03±0.01	0.03±0.01	MS+STD
Benzaldehyde	10.873	0.05±0.01	0.06±0.02	MS+STD
Octanal	11.915	0.22±0.06^b^	0.29±0.09^a^	MS+STD
Benzenacetaldehyde	12.874	0.02±0.01	0.01±0.00	MS+STD
E,2-Octenal	13.19	0.02±0.01	0.03±0.01	MS+STD
Nonanal	14.198	0.20±0.06^b^	0.32±0.11^a^	MS+STD
E,2-nonenal	15.33	0.02±0.10	0.04±0.02	MS+STD
Decanal	15.8893	ND	0.01±0.00	MS+STD
E,E,2,4-Nonadienal	16.0483	ND	0.01±0.01	MS+STD
E,2-Decenal	16.9166	ND	0.03±0.02	MS+STD
E,E, 2,4-Decadienal	17.4938	ND	0.01±0.00	MS+STD
2-Undecenal	18.69	ND	0.02±0.01	MS+STD
Alcohols				
1-penten-3-ol	3.067	0.01±0.00	ND	MS
4-amino-1-hexanol	3.302	0.22±0.03	ND	MS
1-Pentanol	4.7849	0.13±0.03	0.15±0.09	MS+STD
1-Hexanol	8.0627	ND	0.05±0.03	MS+STD
1-Heptanol	11.112	0.01±0.01	0.02±0.01	MS+STD
1-Octen-3-ol	11.356	0.08±0.04	0.07±0.03	MS+STD
2-Ethyl-1-hexanol	12.588	0.03±0.01	ND	MS
1-Octanol	13.1569	ND	0.02±0.01	MS+STD
Sulfur and nitrogen compounds				
Methanethiol	1.5234	ND	0.01±0.01	MS+STD
Carbon disulfide	1.862	0.01±0.00	0.01±0.00	MS+STD
2,5-Dimethyl pyrazine	9.558	0.02±0.01	ND	MS+STD
4-Methylthiazole	11.475	0.20±0.03	ND	MS+STD
2-Ethyl-3,5-dimethyl-Pyrazine	13.575	0.03±0.01	ND	MS+STD
Hydrocarbons				
Toluene	4.7161	0.01±0.00	0.07±0.05	MS+STD
Ethylbenzene	7.7503	ND	0.27±0.45	MS+STD
1,3-dimethyl benzene	7.982	0.01±0.00	ND	MS+STD
Xylene	7.9939	0.08±0.03	0.17±0.14	MS+STD
2,4-Dimethylhexane	13.029	0.03±0.01	ND	MS
Benzoic acid	15.433	0.06±0.02	ND	MS+STD
Dodecane	15.7622	ND	0.01±0.00	MS+STD
Tetredecane	19.262	ND	0.01±0.00	MS+STD

WHD, Woori Heukdon; LYD, commercial ([Landrace×Yorkshire] ♀×Duroc ♂) pig; ND, not detectable; STD, standard.

a,b Means within a row with different superscripts are significantly different (p<0.05).

**Table 6:** Mean values (7-point scale) for sensory properties of shoulder butt and belly of WHD and LYD pigs under identical feeding conditions

Items	LYD	WHD	LYD	WHD
	
	Shoulder butt		Belly	
Color^1)^	4.98±0.83^b^	5.61±0.85^a^	5.13±0.78	5.27±0.84
Flavor	5.07±0.87^b^	5.58±0.77^a^	5.29±0.82^b^	5.79±0.64^a^
Juiciness	5.13±0.55^b^	6.08±0.72^a^	5.33±0.69^b^	6.05±0.68^a^
Tenderness	4.93±0.89^b^	5.82±0.65^a^	4.90±0.93^b^	5.76±0.63^a^
Acceptability	5.28±0.72^b^	5.82±0.66^a^	5.38±0.77^b^	5.85±0.57^a^

The mean values were calculated using 7-point scale.

WHD, Woori Heukdon; LYD, commercial ([Landrace×Yorkshire] ♀×Duroc ♂) pig.

1) Overall color of fresh meat.

a,b Means in the same primal cut within a row with different superscripts are significantly different (p<0.05).

## References

[1] Agriculture and Horticulture Development Board (AHDB) (c2020). The Pork Market in Korea.

[2] Kim SG, Bae HH, Son JY, Shin JS, Ha GH (2020). The trends of consumption of pork meat in Korea 2019 NIAS, RDA, Republic of Korea.

[3] Chung HY, Ko MS (2006). Characteristics and application of native pigs in Jeju South Korea. Soc Cheju Stu.

[4] Jin SK, Kim CW, Song YM (2001). Physicochemical characteristics of longissimus muscle between the Korean native pig and landrace. J Food Sci Anim Resour.

[5] Cho SH, Park BY, Kim JH (2007). Carcass yields and meat quality by live weight of Korean native black pigs. J Anim Sci Technol.

[6] Kim GW, Kim HY (2017). Effects of carcass weight and back-fat thickness on carcass properties of Korean native pigs. J Food Sci Anim Resour.

[7] Kim GW, Kim HY (2018). Physicochemical properties of M. longissimus dorsi of Korean native pigs. J Anim Sci Technol.

[8] Muhlisin, Panjono, Lee SJ, Lee JK, Lee SK (2014). Effects of crossbreeding and gender on the carcass traits and meat quality of Korean native black pig and Duroc crossbred. Asian-Australas J Anim Sci.

[9] Patience JF, Rossoni-Serão MC, Gutiérrez NA (2015). A review of feed efficiency in swine: biology and application. J Anim Sci Biotechnol.

[10] Patience JF, Patience JF (2012). The influence of dietary energy on feed efficiency in grow-finish swine. Feed Efficiency in Swine.

[11] Whittemore CT (1993). The science and practice of pig production.

[12] Schinckel AP, de Lange CFM (1996). Characterization of growth parameters needed as inputs for pig growth models. J Anim Sci.

[13] Hong JS, Lee GI, Jin XH, Kim YY (2016). Effect of dietary energy levels and phase feeding by protein levels on growth performance, blood profiles and carcass characteristics in growing-finishing pigs. J Anim Sci Technol.

[14] Wood JD, Enser M, Fisher AV (2008). Fat deposition, fatty acid composition and meat quality: A review. Meat Sci.

[15] Soladoye OP, Uttaro B, Zawadski S (2017). Compositional and dimensional factors influencing pork belly firmness. Meat Sci.

[16] Ngapo TM, Martin JF, Dransfield E (2007). International preferences for pork appearance: II. Factors influencing consumer choice. Food Qual Prefer.

[17] Hoa VB, Seol KH, Seo HW (2021). 2021. Meat quality characteristics of pork bellies in relation to fat level. Anim Biosci.

[18] Fortin A, Robertson WM, Tong AKW (2005). The eating quality of Canadian pork and its relationship with intramuscular fat. Meat Sci.

[19] Malgwi IH, Halas V, Grünvald P, Schiavon S, Jócsák I (2022). Genes related to fat metabolism in pigs and intramuscular fat content of pork: A Focus on Nutrigenetics and Nutrigenomics. Animals.

[20] (c2018). Korea Institute of Animal Products Quality Evaluation [KAPE].

[21] (2018). Korean Pork Cutting Specification.

[22] Hoa VB, Seol KH, Seo HW (2021). Investigation of physicochemical and sensory quality differences in pork belly and shoulder butt cuts with different quality grades. Food Sci Anim Resour.

[23] Hoa VB, Song DH, Seol KH (2022). Half-castration is a newly effective method for increasing yield and tenderness of male cattle meat. Anim Biosci.

[24] Meilgaard M, Civille G, Carr B (1991). Sensory evaluation techniques.

[25] Cho YS, Park BY, Lee JM, Lee SK (2005). Comparison of carcass and meat quality characteristics between Korean native black pigs and commercial crossbred pigs. Korean J Food Sci Anim Resour.

[26] Ali M, Baek KH, Lee SY (2021). Comparative meat qualities of boston butt muscles (M. subscapularis) from different pig breeds available in Korean market. Food Sci Anim Resour.

[27] Kim DW, Kim KH, Hong JK (2014). Comparision of carcass characteristics, meat quality, and fatty acid profiles between Duroc and corssbred pigs (Duroc × Korean native pig). Korean J Agric Sci.

[28] Kang HS, Seo KS, Kim KT, Nam KC (2011). Comparison of pork quality characteristics of different parts from domesticated pig species. Korean J Food Sci Anim Resour.

[29] Poklukar K, Candek-Potokar M, Lukac NB, Tomažin U, Škrlep M (2020). Lipid deposition and metabolism in local and modern pig breeds: a review. Animals.

[30] Natacha Pena R, Ros-Freixedes R, Tor M, Estany J (2016). Genetic marker discovery in complex traits: A field example on fat content and composition in pigs. Int J Mol Sci.

[31] Urrutia O, Alfonso L, Mendizabal JA, Szablewski L (2018). Cellularity description of adipose depots in domesticated animals. Adipose Tissue.

[32] Wood JD, Nute GR, Richardson RI (2004). Effects of breed, diet and muscle on fat deposition and eating quality in pigs. Meat Sci.

[33] Faucitano L (2018). Preslaughter handling practices and their effects on animal welfare and pork quality. J Anim Sci.

[34] Bocian M, Wojtysiak D, Jankowia H, Cebulska A, Kapelanski W, Migdal W (2012). Carcass, meat quality and histochemical traits of m. longissimus lumborum from Zlotnicka spotted pigs and commercial pigs. Folia Biol (Krakow).

[35] Rosenvold K, Andersen HJ (2003). Factors of significance for pork quality-a review. Meat Sci.

[36] Hoa VB, Cho SH, Seong PN (2021). The significant influences of pH, temperature and fatty acids on meat myoglobin oxidation: a model study. J Food Sci Technol.

[37] Smith SB (2016). Marbling and its nutritional impact on risk factors for cardiovascular disease. J Food Sci Anim Resour.

[38] Jones PJH, Rideout T, Ross AC, Caballero B, Cousins RJ, Tucker KL, Ziegler TR (2012). Lipids, sterols, and their metabolites. Modern nutrition in health and disease.

[39] Department of Health (1994). Nutritional aspects of cardiovascular disease (report on health and social subjects no 46).

[40] Mottram DS (1998). Flavour formation in meat and meat products: a review. Food Chem.

[41] Hoa VB, Cho SH, Seong PN (2020). Quality characteristics, fatty acid profiles, flavor compounds and eating quality of cull sow meat in comparison with commercial pork. Asian-Australas J Anim Sci.

[42] Hoa VB, Ryu KS, Hwang IH (2012). Flavor characteristics of Hanwoo beef in comparison with other Korean foods. Asian-Australas J Anim Sci.

[43] Elmore JS, Mottram DS, Enser M, Wood JD (1999). Effect of the polyunsaturated fatty acid composition of beef muscle on the profile of aroma volatiles. J Agric Food Chem.

[44] Hoa VB, Amna T, Hwang IH (2013). Significant influence of particular unsaturated fatty acids and pH on the volatile compounds in meat-like model systems. Meat Sci.

